# Supplementary material for: Use of a mechanistic growth model in evaluating post-restoration habitat quality for juvenile salmonids
Source: PLoS One. 2020 Jun 24;15(6):e0234072. doi: 10.1371/journal.pone.0234072 (PMC7313756; doi:10.1371/journal.pone.0234072)
Supplement: Attachment — Submitted filename: Response Letter PONE-D-20-02574.docx [file pone.0234072.s003.docx]

Maura Geraldine Chapman, PhD DSc.

Academic Editor
PLOS ONE

RE: PONE-D-20-02574

Dear Dr. Chapman:

We submit for review a revision of PONE-D-20-02574 with gratitude for the opportunity to revise the manuscript according to the comments of the two Reviewers. Both reviewers asked pertinent questions that enabled us to clarify the specific application of our model to fisheries management, but to retain the generality of the model to be applied across taxa. At least one reviewer acknowledged the wide applicability of the model and both reviewers acknowledged the novelty of our approach in both fisheries ecology and restoration ecology. We are appreciative of these recognitions.

We have few general responses – our point-by-point responses are below in the context of the specific reviewer comments. The main general editorial issue is that we prepared the manuscript using LaTeX and therefore we do not have a “Track Changes” version that was requested in the Editor’s response letter. In this response letter, we have indicated specific line numbers that refer to issues raised by the Reviewers and where changes and additions can be found. Unless indicated as line number in the previous draft, these refer to line numbers in the *revised* draft submitted now. We also appended some notes in the PDF of the original submission to indicate where substantial modifications were needed, in keeping with the most substantive Reviewer comments; however, many of the questions involved making edits in several locations and the responses below will provide better guidance to the locations of those.

Finally, in the responses below and in the revised draft, we make reference to two “*in press*” papers from this study system that were still in review at the time of the original submission of this paper. At the end of the responses, we provide links to pre-prints of these two papers if the reviewers and editor find referring to them useful in assessing these revisions.

We look forward to the next review of our manuscript.

Thank you for your consideration,

Carlos M. Polivka, corresponding author, on behalf of:

Joseph R. Mihaljevic and Greg Dwyer

Reviewer #1: This study is a great example of using novel methods to evaluate the age-old question of evaluating restoration effectiveness. I think measuring the growth effects, rather than changes in local abundance, is a great metric that is often not evaluated because other approaches have not worked well, or the mark-recapture data have been too difficult to obtain. Measures of changes in abundance are often discounted because it is assumed that restoration is merely drawing fish from other habitats with no appreciable benefit to the population. However, growth, as the authors mention has correlations with other important demographic parameters. In short, I think this manuscript illustrates a technique that others should and probably will take advantage of when evaluating restoration. For me, as someone unfamiliar with the Entiat River, the management implications are harder to wrap my head around. The authors state that the unrestored reach is nearby, and also has pools, but those pools are smaller.

I always struggle with studies of this type where the comparison is commonly to an unrestored area. I am always wanting to see comparison to a “reference” area, where instead of asking “does restoration improve growth compared to unrestored areas?” you might ask “Is the restoration providing similar growth as a natural wood jam produced pool?” These questions are different but we tend to only see the former, not the latter, and I don’t know why. Maybe there is no natural wood recruitment in the system?

*Response: There is natural wood in the Entiat River, but it occurs primarily in the upper valley basin where nearby land use is less intensive. The study reaches here are in the lower basin where residences and agricultural land dominate the floodplain. One of the intended effects of restoration with the ELJs is to have structures in place on which additional natural wood may recruit and “rack up.” We added a few sentences to the study system description to explain the reasons for reduced wood and rationale for ELJs (LINES 88-92).*

Does the unrestored reach also have wood, or are the pools different in other aspects besides their size? I suppose I was expecting to see this aspect introduced as a testable hypothesis.

*Response: The unrestored study pools did not contain wood in any of the study years (see previous response). Thus, the hypothesis being tested was whether the addition of wood creates habitats in which fish experience higher growth rates relative to movement or use of pools without wood. Our point is that showing that some correlate of fitness (growth) is positively affected is a critical component of restoration ecology and our hope is that others will, indeed take advantage of this approach, as the Reviewer stated.*

Were you expecting that growth would be enhanced by the new pools? If so, why?

*Response: The crux of the study was to demonstrate that restoration should be evaluated with a measurable response in a trait important to fitness in the target species. So, we expected that growth would be a trait for which we could observe a positive effect of restoration (previous and new draft LINES 47-48; previous draft 73-75, new draft 78-79). The hypothesis tested was that fish would experience higher growth in the ELJ-restored pools.*

Might there be a more diverse invertebrate community? Is there more cover allowing for less vigilance among individuals?

*Response: We did not directly measure invertebrate availability for the same reason given the original draft with regard to physical habitat characteristics. It is logistically infeasible to obtain continuous measurements of invertebrate delivery or other physical properties in the field (text added to LINES 157-167). Specific to the Reviewer’s point about aquatic macroinvertebrates, we added text (LINES 496-506) because one of the ecological rationales for in-stream structures such as ELJs is to increase invertebrate production. This also ties in with the Reviewer’s question about vigilance. Pools with cover are usually postulated to offer the opportunity for more vigilance and are predicted to be used by individuals that require less energy and thus take fewer foraging risks into shallower, faster flowing habitats that typically have higher delivery rates of invertebrates (see citations on LINES 501-502, including a related “in press” publication from this study system). In that added text, we acknowledge that invertebrate production may be mechanism behind our observation of higher growth and that our results motivate further study of invertebrate availability.*

You mention that the densities are similar among restored and unrestored reaches. Is there possibly then, another aspect of the allometry of pool size that provides thresholds of benefit at similar volumetric density? I would think so. It does appear that the interspecific interactions are very important as the authors suggest, and there is a clear residence time component. I think what I am left wondering is if the unrestored reach had larger pools, would we see a difference, or is it the wood, irrespective of pool size that provides the benefit? You mention that work with specific environmental covariates yielded nothing conclusive, but form a restoration perspective, is it the pools or the wood or does that matter because you cannot get large pools without the wood?

*Response: The Reviewer’s premise statement is incorrect. Lines 380-382 and Table 1 unequivocally indicated that density of both species is higher in restored pools with all years combined. For steelhead, some individual study years had similar densities among habitats (and no growth differences in those years). To the question of pool size, the density comparisons used here were taken from Polivka et al. (2015) for the first four study years and the same method was used to obtain the 2016 density comparison. That reference and Polivka and Claeson (in press) show that, after correcting for area, ELJ pools still have higher density than unrestored pools. This clarification should clear up confusion from LINES 98-102 that both types of pools do increase in density with size, even at the same rate, but overall density is higher in restored pools. Because the pools have higher density regardless of area, there is another mechanism at work – possibly invertebrate production as discussed in the previous response.*

Unrestored pools are smaller, and there are no recapture records of Chinook I those habitats. Could it be that the unrestored habitat is really representative of a smaller transient class of fish that is moving downstream, rather than indicative of residence in that habitat?

*Movement and transience were a persistent issue and, in the case of Chinook, the transient nature of the fish observed there is why we compared all transient fish (regardless of pool of original capture) with those that resided (longer-term) in restored pools. Recall, however, that the transient fish are a combination of fish captured in both restored and unrestored habitat, which makes the analysis more conservative (see LINES 150-154). Furthermore, there is separate evidence that fish in unrestored pools do have short-term residency in the pools (added citation Polivka, in press), just not long enough for measurable growth (LINES 82-84, 130-131).*

L88: is this a reference? “beechie2005classification”

*Response: Yes, a syntax error in the LaTeX document programming code. Fixed.*

Figure 1 could include a little more context. Can you provide a scale bar?

*An improved version of Figure 1 is included in this draft, while noting that the other Reviewer suggested that it was unnecessary. We leave it to the Reviewers and Editor to determine whether this improvement warrants keeping the Figure, or whether a modified figure would be better.*

Figure 3. Is this just a sample “hypothetical” plot? Why not label the arrow with R-hat to make it work with the caption.

*Response: Yes, this is a hypothetical plot. However, the arrow only represents one of the R values used in calculating the R-bar (which is the average of values for each habitat type). We added the letter R in parentheses after “Residual” to be more congruent with the figure caption and edited the caption slightly for clarity.*

Reviewer #2: Review of PONE-D-02574

This paper presents a mechanistic growth model used to evaluate the ecological effectiveness of river habitat enhancement on threatened juvenile salmonid populations in Washington state. Most habitat restoration/enhancement studies rely on density estimates to determine whether a conservation action achieved its objective even though density is a misleading indicator of habitat quality. The authors applied the model using growth data collected from mark-recapture of juvenile fish in a river reach where large wood was added and compared these data to an unrestored reach. They also compared growth results to density estimates to determine congruence in how they reflected the response to habitat enhancement. Model parameters not only described the rate of individual growth but also the timing of increases. The model was also spatially explicit accounting for habitat differences. The authors suggest that these characteristics make it widely applicable to different taxa.

General comments: I found this article well-written and logically presented. Moreover, the topic is timely as billions of dollars have been spent to enhance habitat degraded by human activities but approaches to assess the effectiveness of these actions are limited to mostly monitoring changes in density, which is not a reliable indicator of habitat quality.

I would suggest the authors use “habitat enhancement” rather than habitat restoration as the project they evaluated did not restore the process of wood loading to river channels. Instead, the project enhanced wood abundance in the river.

*Response: The Reviewer’s point is technically true and we have made some adjustments to the text to be clear that wood was added to “enhance” habitat. However, other published papers from this study system refer to the efforts as habitat restoration, augmenting pools with wood, which restores and/or enlarges pools, increases cover, etc. We incorporated the reviewer’s suggestion of using “enhancement” by linking the term “ELJ-enhanced pools” with “restored pools” (LINES 95-96). For shorthand, we use “ELJ-pools” if “ELJ-enhanced pools” was specified earlier in an associated paragraph. Edits were made throughout to incorporate this modification.*

Specific comments:

Line 36: This is the first time we learn about salmonids, which are the main players in the paper. More details on what they are and why they are of interest is needed. Also, I would suggest introducing them earlier in the introduction.

*Response: Because this journal is intended for a more general audience, we focus on the quantitative application to restoration ecology, as opposed to an article written for an ichthyological/fisheries-oriented journal where we would focus the introduction on fish. Nevertheless, the Reviewer’s point is well taken, so we reorganized the Introduction somewhat to maintain the quantitative focus, but to introduce the study organism a little earlier (LINE 17).*

Figure 1. I’m not sure how much the map adds to the paper.

*The other Reviewer suggested an improvement to the map, which we made for this draft and we leave it to the Reviewers and Editor to decide whether the map should be kept.*

Fish marking: What about fish movement? This is an open system where fish are moving among habitats. The authors need to address this complication to their story.

*Response: We previously addressed movement differences as the rationale for different approaches to analysis of Chinook and steelhead data because Chinook are very mobile and recaptures on a time scale needed for growth were not possible in the unrestored pools (previous draft LINES 130-133, 232-233). We have added text in several sections and retained that previous text to more thoroughly address how movement is integrated with the interpretation of our data (LINES 54-57, 82-84, 141-145, 255-257, 431-435, 445-450, and 473-474).*

Local density: Why is local density not part of the model?

*Response: Because of movement considered in the previous point, density likely changed as often as daily. As with all potential correlates of growth, the complications of accounting for changes in each parameter between the marking date and recapture date. Our rationale is based in the statement made on LINES 29-31 that growth models serve when there are multiple ecological inputs. We return to this point about covariates and provide further explanation in the Data Analysis section (LINES 157-168). We considered density separately in Table 1. Thus we show that despite higher density in restored pools, there is a growth benefit for Chinook in three of five individual study years and for all years combined. We added this last point in the Discussion as well (LINES 519-521).*

Line 161: I would like to see an explicit definition of a (alpha) here. We don’t learn what it is in detail until later in the paper.

*Response: We added text to LINES 182-184 to explicitly define alpha-hat.*

Line 388-389: Provide references for this statement.

*Response: We added the previously cited references Tarkan et al. (2016), Street et al. (2015), and, specific to salmonids, Achord et al. (2007).*

Any ideas of why there were annual differences in response (e.g., Figure 8)?

*Response: Annual differences in the response are indicated in all figures. At this point, we have found that habitat enhancement is associated with higher growth rates and this serves as motivation for further study of specific inputs into growth (see LINES 166-168). Specific to Figure 8, it confirmed the model output indicating years in which growth differences among habitats were observed, which was the key point of that analysis. The other important point is that growth may not support the observed density difference (e.g., 2012). We stated that steelhead in this system have been repeatedly found to have a more generalist pattern of habitat use in this and other study systems (LINES 461-467; Everest and Chapman, 1972; Young et al., 2004; Polivka et al., 2015; Polivka and Claeson, in press), which may mean less frequent differences in growth among habitats.*

**Preprints of “in press” references:**

Polivka, C.M. and S.M. Claeson. Beyond redistribution: In-stream habitat restoration increases capacity for young-of-the-year salmon and trout in the Entiat River, WA. *North American Journal of Fisheries Management.*

[https://www.biorxiv.org/content/10.1101/665604](https://www.biorxiv.org/content/10.1101/665604v2)

Polivka, C. M. Habitat affinity and density-dependent movement of juvenile salmonids in response to restoration. *ECOSPHERE*.

[https://www.biorxiv.org/content/10.1101/712935](https://www.biorxiv.org/content/10.1101/712935v2)
